# Exploring perceptions, barriers, and enablers for delivery of primary ear and hearing care by community health workers: a photovoice study in Mukono District, Uganda

**DOI:** 10.1186/s12939-020-01158-8

**Published:** 2020-05-07

**Authors:** James O’Donovan, Allan S. Namanda, Rebecca Hamala, Niall Winters, Mahmood F. Bhutta

**Affiliations:** 1 Department of Education, The University of Oxford, Norham Gardens, Oxford, OX2 6PS UK; 2 Division of Research and Health Equity, Omni Med Uganda, Makata, Mukono District, Mukono, Uganda; 3 Department of ENT, Brighton and Sussex University Hospitals NHS Trust, Brighton, UK

**Keywords:** Hearing loss, Ear disease, Community health workers, Photovoice, Participatory visual methods

## Abstract

**Background:**

Hearing loss is a prevalent but neglected disease, especially in low- or middle-income countries. The role of Community Health Workers (CHWs) to deliver primary ear and hearing care has been explored in several studies from a technical standpoint, but understanding perceptions, barriers, and enablers of such an approach from the perspective of CHWs themselves through a health equity lens has been less well documented.

**Methods:**

This qualitative study used photovoice to explore the views and experiences of CHWs in the Seeta Nazigo Parish of Mukono District in the delivery of ear and hearing care in the community. CHWs were trained in ear and hearing care, and provided with digital cameras to capture photographs related to their work in the community over the following 3 months. Individual interviews regarding the photographs were held at the end of each month, in addition to one focus group discussion. A community workshop was convened at the end of the study to display the photos. Thematic analysis of photographs was conducted using Braune and Clarkes six-step framework. We also used the data to explore potential roles for key stakeholders in primary ear and hearing care, and how photovoice may facilitate their engagement.

**Results:**

13 CHWs participated in the study. Several themes were generated from analysis. CHWs perceived a high burden of ear and hearing disorders in their community and recognised the role they could play in tackling that burden. Potential barriers identified included a lack of equipment, training, and supervision of CHWs; logistical, financial, or psychological barriers to community participation; and the widespread use of traditional medicine. CHWs identified roles for the government and NGO bodies to enable and support delivery of ear and hearing care in the community. The community workshop was a useful method to engage key stakeholders in this topic.

**Conclusions:**

Photovoice is a powerful method to capture issues affecting CHWs. Here it was used to identify a number of perceptions, barriers and enablers to the delivery of ear and hearing care. Our results may inform future strategy in the field of ear and hearing care, and the potential use of photovoice to enact sociocultural change.

## Background

Hearing loss is estimated to affect 6.1% of the global population, with the greatest burden of disease in countries defined as low- or middle-income (LMIC) [1]. Yet, despite an estimated global cost of $750 million dollars annually [2], hearing loss is considered a neglected public health issue [3].

The World Health Organisation (WHO) strategy to tackle this issue includes the use of community health workers (CHWs) to take on tasks related to diagnosis and management of ear and hearing disorders [4], particularly where relevant specialist human resources are sparse, such as in LMICs [5]. It has been suggested that community health programmes can help to improve the equity of care, especially in low-income settings, by bringing healthcare services close to the community and thus increasing accessibility, acceptability and reducing gaps in quality of care [6, 7]. For the purpose of this study, we use Whitehead’s definition of health equity, which is “equal access to available care for equal need; equal utilisation for equal need; and equal quality of care for all” [7].

A recent systematic scoping review identified 38 studies where CHWs had been deployed in task sharing models to address ear and hearing disorders [8], in particular to undertake or increase community participation in screening (using whispered voice tests, noisemakers for neonatal screening, automated audiological tests, and/or otoscopy), as well as a handful of descriptive studies on CHWs in management interventions such as ear washout (for wax or infection), applying antibiotic ear drops, or fitting hearing aids [9–11]. However, no literature evaluated the long-term outcomes of CHWs in delivering ear and hearing care, or explored the potential barriers and incentives to the success of such programmes.

In other contexts, CHWs have been deployed in a variety of roles and tasks. This includes diagnosis of disease (e.g. screening for breast cancer or testing for malaria), treatment of disease (provision of antimalarial medication or vaccination), health education (for example around family planning or breastfeeding), recording data (for example on stocks of medical supplies), or providing psychosocial support (for example instigating support groups for people with HIV) [12]. In these studies threats to the performance, motivation, and job satisfaction of CHWs include over-burdening of CHWs with tasks, inadequate training and ongoing support, poor logistical support, inadequate remuneration, and a lack of integration into health systems [12].

In this study we took a participatory approach towards exploring potential perceptions, barriers and enablers to a CHW led ear and hearing care programme established in Mukono District, Uganda. This was achieved through the use of photovoice, a participatory visual methodology where users are provided with cameras to capture photographs relevant to the topic of interest [13]. Photovoice has three main aims: to enable individuals to reflect their communities strengths and concerns through visual images, to engage in and promote critical dialogue around the images to raise awareness of a topic of social importance, and to reach policy makers [14]. The little existing literature on the use of photovoice with CHWs, suggests it is a potentially powerful method to encourage reflective practice, understand complex health issues, and identify key issues in the community to leverage social action [15]. It can also help to provide rich, multidimensional data on the lived experience of the participant, which may not be captured through traditional, researcher led, methods of enquiry [16].

Here we view the resulting themes related to the perceptions, barriers, and enablers of a CHW led ear and hearing care programme through a health equity lens. We also explore potential roles for key stakeholders in the delivery of ear and hearing care at the community level, and evaluate how photovoice may facilitate their engagement and participation.

## Methods

### Study design and setting

This exploratory qualitative participatory study took place between April and July 2019, in Seeta Nazigo Parish, a rural area located in the Nakisunga sub-county of Mukono District, central Uganda. Mukono District is part of the Buganda Kingdom, the largest of the traditional kingdoms in Uganda, with a population of 6 million, comprising approximately 17% of the country total [17]. The local population are referred to as Baganda, and the primary language of the region is Luganda.

The Nakisunga sub-county has a population of 48,000 people [18], with a primary occupation of subsistence farming. There is one government-only funded health centre in the sub-county called Seeta Nazigo Health Centre III (SN HCIII), which employs nine full time staff, including three nurses, two midwives, two lab technicians, a data assistant and a clinical officer [19]. The health facility staff are supported by a team of CHWs, known locally as Village Health Team members (VHTs). CHWs in Uganda are expected to carry out multiple tasks relevant to the provision of primary health care, including conducting home visits, mobilisation of communities, general health promotion and education, addressing maternal and child health issues, and community follow up of recently discharged or chronically ill patients [20, 21].

The CHWs in Mukono District are supported and trained by several non-governmental organisations (NGOs), including Living Goods, Healthy Entrepreneurs, Bangladesh Rural Advancement Committee (BRAC), and Omni Med. The NGOs support the work of the Mukono District Health Office (DHO) by training and supporting CHWs, but the nature and extent of support varies by NGO partner, resulting in an inconsistent landscape of primary health care delivery. The NGO supporting the largest number of CHWs in this region is Omni Med - estimated at 1250.

### Study participants

The participants were 13 volunteer CHWs selected from the Seeta Nazigo Parish, all of who were primarily supported by Omni Med. All were already enrolled in the larger project exploring the delivery of primary ear and hearing care to this region (*see Study Background and Context sub-section for further details*).

Participants were selected purposively, with the aim of recruiting CHWs with a range of ages and a balanced number of males and females. We initially aimed to recruit CHWs with a range of experience, but this proved not possible because no new CHWs had been recruited since the initial recruitment drive between 2008 and 2010. We only recruited CHWs who were over the age of 18, actively practicing, and willing and consenting to participate in the study. We did not exclude on the basis of age, language, gender, sex or tribe [22].

A total of 13 CHWs participated in the photovoice study, including 7 females and 6 males, all of who had served between 8 and 11 years as a CHW. A summary of demographic details can be found in Table 1.

**Table 1:** Community Health Worker participant demographics and characteristics

CHW	Sex	Age	Years of Education	Highest level of education	Job	Number of years as CHW	Number of households served
CHW1	F	50	9	Some secondary	Farmer	8	50
CHW2	F	42	12	Some secondary	Farmer	8	38
CHW3	F	44	9	Some secondary	Farmer	8	30
CHW4	M	45	7	Some primary	Builder and farmer	8	120
CHW 5	F	31	13	Completed secondary	Farmer	10	105
CHW6	M	67	8	Some primary	Farmer	8	35
CHW7	F	34	9	Some secondary	Farmer	8	68
CHW8	M	40	11	Some secondary	Farmer	8	35
CHW 9	M	53	11	Some secondary	Farmer	10	35
CHW 10	F	50	13	Some secondary	Shop attendant and farmer	8	100
CHW 11	F	46	13	Some secondary	Farmer	10	100
CHW 12	M	39	13	Completed secondary	Farmer	11	50
CHW 13	F	42	12	Some secondary	Farmer	8	38

Details and demographics for the 13 CHWs who participated in this study.

### Study background and context

This study was embedded in a larger project exploring training CHWs in the delivery of primary ear and hearing care in the community. That project arose as a result of an earlier participatory action research project, where CHWs in this region requested such training due to the high burden of ear and hearing disease they encountered in the community. A detailed description of how this larger project was established, and an evaluation of the training and outcomes will be described in a later publication; however to contextualise this particular study we will provide a brief overview here.

CHWs were trained in primary ear and hearing care services through a four-day training course, led by a team of Ear, Nose and Throat (ENT) surgeons and specialists in community ear care from the UK, Australia, and Uganda. Training material was developed and modified from the WHO Primary Ear and Hearing Care Training Manuals [23], and focussed on the recognition of common ear problems in the community, history taking, examination, and basic management skills (such as ear washout, dry mopping, and instillation of antibiotic ear drops). The training comprised lecture based teaching and hands-on training in practical skills. Following the initial training, CHWs were assessed in examination, case management, and treatment skills through an Observed Structured Clinical Examination. All 13 CHWs who participated in this study were deemed to be safe and competent in performing these tasks. The CHWs were then deployed in the community to provide basic ear and hearing care services to the households they were responsible for, and were aided in this task through the provision of a low-cost smart phone to contact their supervisors, equipment (including a low-cost otoscope and equipment for basic treatment), and a monthly stipend for transport.

This particular photovoice study arose from a direct request by one of the CHWs. Eight of the local CHWs had previously participated in a photovoice project exploring the challenges they faced in their role as volunteer CHWs [19]. One of these CHWs approached the study principal investigator (PI) in March 2019, suggesting that the CHWs undertake a photovoice project to explore their experiences of delivering primary ear and hearing care in the community. As a result all 13 CHWs involved in the larger study were called to a meeting at SN HCIII, facilitated by the study research team, to discuss the proposal. CHWs discussed the particular elements they wished to explore as part of the photovoice assignment, as well as the frequency of meetings to discuss photographs. It was decided that the remit of the project should be broad, so as to capture all potentially important or relevant experiences and factors in the delivery of community-based ear and hearing care, including the challenges faced. It was also agreed that individual interviews to discuss photographs should take place once a month.

### Implementation team and partners

The direct implementation team consisted of the PI (a UK trained physician who was resident in the community for 12-months) and two Ugandan research assistants (RA) who hold Bachelor level degrees and are fluent in English and Luganda.

The study was also facilitated by several partners. The study PI had an on-going relationship with Omni Med who granted permission to recruit and work with the CHWs they supported, as well as providing in-kind support, such as access to vehicles and accommodation for the duration of the study. From the outset the study team involved the Mukono DHO to ensure that the delivery of a primary ear care service aligned with the overall strategic health objectives of the district. This process of engagement involved meeting and briefing several key members of the DHO team, inviting them to conduct monitoring visits, and providing project updates on a quarterly basis. At the beginning of the project the study team also briefed the principals of local Primary and Secondary Schools, religious leaders, the local health centre and local government representatives. Finally, a team of Ugandan ENT doctors were involved in case there was a need to refer community members for expert opinion or intervention.

### Training workshop

To familiarise the CHWs with the photovoice method, a one-day training workshop was held at SN HCIII led by the study research team. All 13 CHWs attended and were trained on the process of obtaining informed consent for capture of photographic images, as well as in the use of the digital cameras. The CHWs who had taken part in the earlier photovoice study were familiar with the digital cameras, and as a result assisted the CHWs without previous experience.

### Materials

CHWs were supplied with a ZoomK 2.7-in. display 18-megapixel digital camera (ZoomK, China), a note book, pens, and consent forms. CHWs also received a small monetary reimbursement ($5 USD) to assist with transportation costs each time they attended a meeting.

### Photovoice assignment

Commencing in April 2019, CHWs were instructed to capture images related to their role in delivery of primary ear and hearing care in the community, but were also given permission to use the cameras for their own personal use if they so wished. After one month CHWs were called to SN HCIII to discuss their photographs in individual interviews conducted in Luganda by the RA’s. Prior to commencing interviews, photographs were transferred from the camera’s memory card to a laptop. The RA asked the CHWs general questions around how they had perceived the project and challenges they faced, before beginning discussion around the photographs. The RA went through individual photographs captured by the CHW, and undertook an in-depth discussion for each photograph, relevant to the topic of interest. The RA determined if photographs were relevant to the topic of delivering primary ear and hearing care in the community by directly asking the CHW if this was the case. For example, personal photographs depicting wedding functions were considered not relevant and were therefore not discussed in detail.

Discussions were framed around the ‘SHOWED’ mnemonic; a commonly deployed method in photovoice studies [13], which consists of five questions;

1. What do you **S**ee here?
2. What is really **H**appening here?
3. How does this relate to **O**ur lives?
4. **W**hy does this condition **E**xist?
5. What can we **D**o about it?

Interviews were recorded using a handheld audio recorder and translated and transcribed into English in a Microsoft Word document within 24 hours of interview by the same RA who conducted the interview. Any words in Luganda which did not translate directly into English were kept in their original form, and an approximate translation included in parenthesis directly after the word or phrase. The translated transcript was then checked against the original audio recording for accuracy by a different RA. This process of individual interviews was conducted once a month, for three months, until data saturation.

After three rounds of individual interviews, a focus group discussion was held with all 13 CHWs. All photographs relevant to the subject of primary ear and hearing care were printed and discussed by the group of CHWs to explore their perceptions of colleagues’ photographs. All photographs were printed in order to avoid selection bias by the research team. At the end of group discussion, similar photographs were grouped into clusters to guide final thematic analysis. Upon completion of the study CHWs were asked if they wished to keep a copy of any personal photographs they had captured as a gesture of goodwill.

### Community workshop

A one-day workshop was held at the Omni Med offices in August 2019, upon completion of the photovoice assignment to celebrate the achievements of the CHWs, and to share the photographs with the wider community and key stakeholders. Invitations were sent to key stakeholders identified by the CHWs during the focus group discussions. Invited stakeholders included members of the District Health Office, NGO facilitators, CHWs, local chairpersons, members of the local and national press, members of the academic community and health centre officials, and community members. Transport refunds were offered to attendees in line with national published rates.

**Fig. 1:**
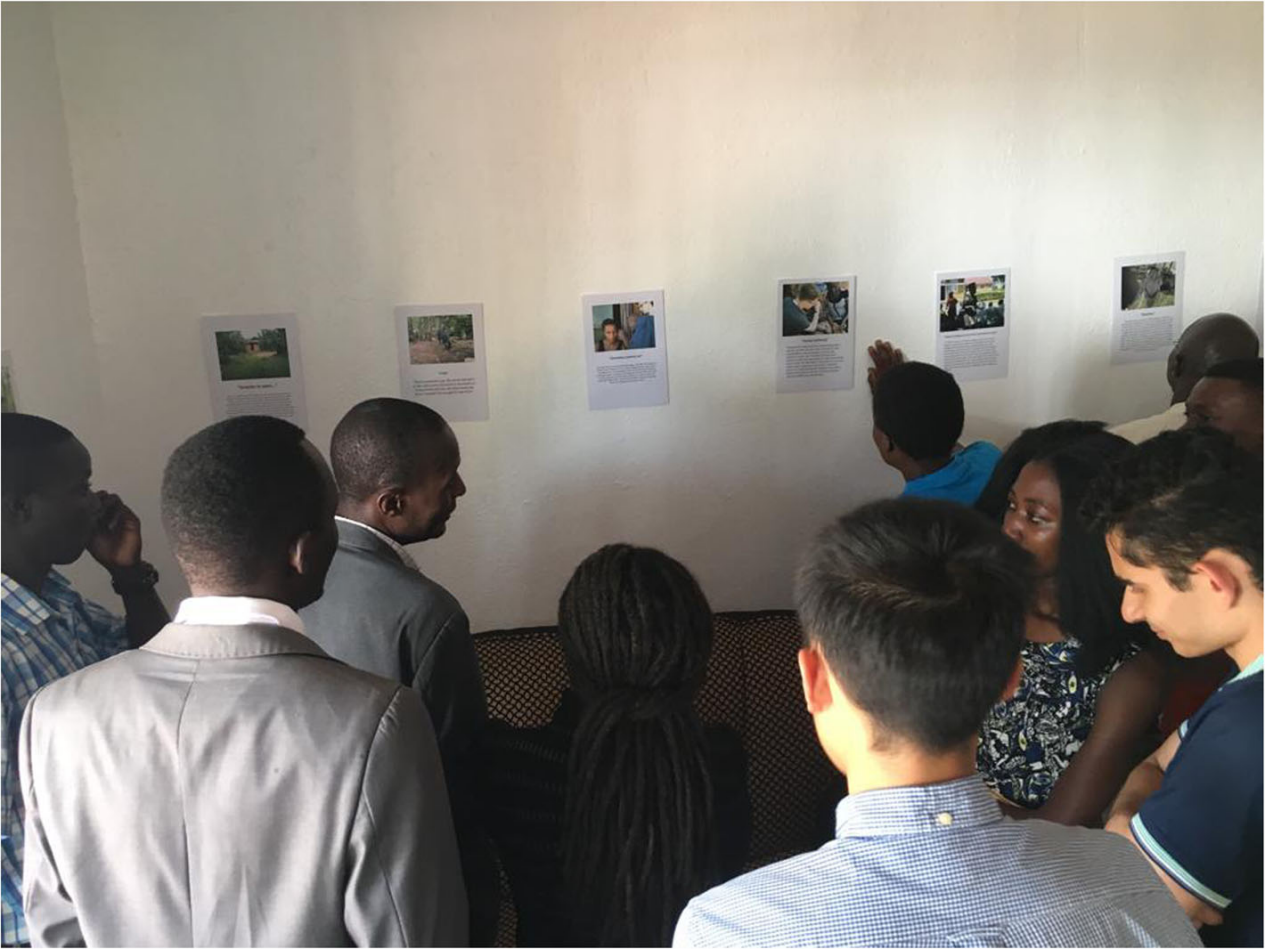
Photo display at Community Workshop. Photographs captured by the CHWs are displayed at a Community Workshop. In this photograph, a female CHW (blue t-shirt) stands by her photographs and explains to the workshop participants the meaning behind the photographs

The workshop consisted of short speeches lasting 10–15 min from each of the key stakeholders as to what the project meant to them, how it had been important, and areas for improvement. Participants were then invited to view the photographs captured by the CHWs as part of a gallery style display. Photographs were grouped according to the CHW who took them to allow the CHW the opportunity to stand near their photographs and discuss what they represented with attendees (Fig. 1). The workshop concluded with an open-floor discussion on how any issues that had been identified should be taken forward, and who should be responsible for this. Finally, there was a certification ceremony recognising the work of the CHWs.

Following the conclusion of the workshop participants were invited to complete an anonymous feedback questionnaire regarding their satisfaction and opinion of the workshop. The questionnaire was adapted from previously validated questionnaires and provided in both English and Luganda, according to the preference of the individual (*see Supplementary Material*). Four questions were included on the participants perceptions of the workshop with answers on a 5-point Likert scale, ranging from 1 (Extremely Unlikely/Very Bad/Not Useful at all) to 5 (Extremely Likely/Very Good/Extremely Useful), as well as open ended questions on how they felt the workshop could be improved. The workshop was filmed by a journalist from the major Luganda speaking national television station, Bukedde TV.

We summarise the steps taken in the conduct of this study in Table 2, which future studies may wish to modify and adapt to their own context (see Table 2).

**Table 2:** Summary of steps involved in our Photovoice study

Stage	Description of Stage
**1 – Ethics approval**	We applied for ethical approval from both our host institution and the local ethics board(s) in Uganda in order to conduct the photovoice study. We engaged regularly with the review boards to update them with any changes to the project protocol.
**2 - Selection of participants**	Once ethical approval was granted we selected participants in a participatory manner, by consulting with key stakeholders. We used purposive sampling to select a diverse range of participants.
**3 – Topic selection**	We held a joint meeting with CHWs and study facilitators to decide upon the topic that was to be the central focus of the project. In the case of this study, CHWs selected to explore experiences of delivering primary ear and hearing care in the community.
**4 – Training of participants in the use of cameras and obtaining informed consent.**	This stage involved technical training of CHWs in the use of cameras, as well as how to obtain informed consent.
**5 - Photograph elicitation and subsequent interviews**	This stage was conducted in a cyclical manner and completed once a month, over a three month period until data saturation.
**6 - Sorting and grouping of photographs**	Photographs were grouped and sorted into themes. This was first done individually and then as a group.
**7 – Dissemination and sharing of findings**	Findings were shared with the wider community, through a community workshop.

The key stages involved in our photovoice study.

### Data analysis

Once all of the interviews had been transcribed, formatted for stylistic consistency, and checked for accuracy, they were exported and saved into NVivo (Version 12 for Mac) qualitative analysis software [24]. Following initial data cleaning and checking, the PI and a study RA conducted thematic analysis of individual interviews and accompanying photographic images using Braun and Clarke’s six-step framework [25]. Initial codes were generated relevant to the subject of ear and hearing care delivery in the community, and then developed using an open-coding framework, meaning that they were generated and modified throughout the process of reading the transcripts. Each set of codes was generated by each researcher independently, and then discussed jointly before determining a set of unified codes based on the initial analysis. The second RA then checked a random sample of quotes independently to ensure they agreed with theme assignment.

Finally, descriptive statistics were used to analyse the evaluation questionnaires completed at the end of the community workshop.

## Results

### Photovoice study: photograph characteristics

Over the three-month period, CHWs took a total of 189 photographs, with 88 of these (and the discussion around them) deemed relevant to the subject of primary ear and hearing care delivery in the community. On average CHWs took a total of 14 photographs each over the three month period, with a range between 2 and 24. Although there were some minor technical difficulties in using the camera (such as issues with knowing how to delete photos) these mainly occurred at the beginning of the project and CHWs who had been involved in a previous photovoice study were able to assist their peers in resolving these challenges. No cameras were damaged, lost, or stolen over the course of the project.

### Results of thematic analysis of photovoice individual interviews and focus group discussion

We now present the themes developed through the thematic analysis of the photos and resulting interview transcripts. For each theme we describe or display at least one photograph and provide the related caption(s), followed by the participant’s study number. While all service-related photographs were useful for discussion, caption-writing, and community display, some were not of sufficient quality for inclusion in the paper. We hope that the simple descriptions will suffice in providing a context for the associated caption.

### Theme 1: capturing images highlights burden of disease and acts as an aid for CHWs to document and discuss cases with their supervisors

The majority of CHWs used the study as an opportunity to capture photographs of members of the community with ear disorders in order to highlight the burden of disease, document their work, and to serve as reminders for cases they wished to discuss with their supervisors. The photographs helped to reflect the high and diverse burden of ear problems in the community, including wax, chronic suppurative otitis media, hearing loss, foreign bodies, and ear trauma. Most commonly the CHW captured a photograph of an individual community member, with the resulting discussion focussing on the ear disorder or problem they faced. For example one photograph depicted the ear of a child. In the resulting discussion of the photograph the CHW stated:

*“I found a small bicycle part from her ears.”* (CHW 12).

**Fig. 2:**
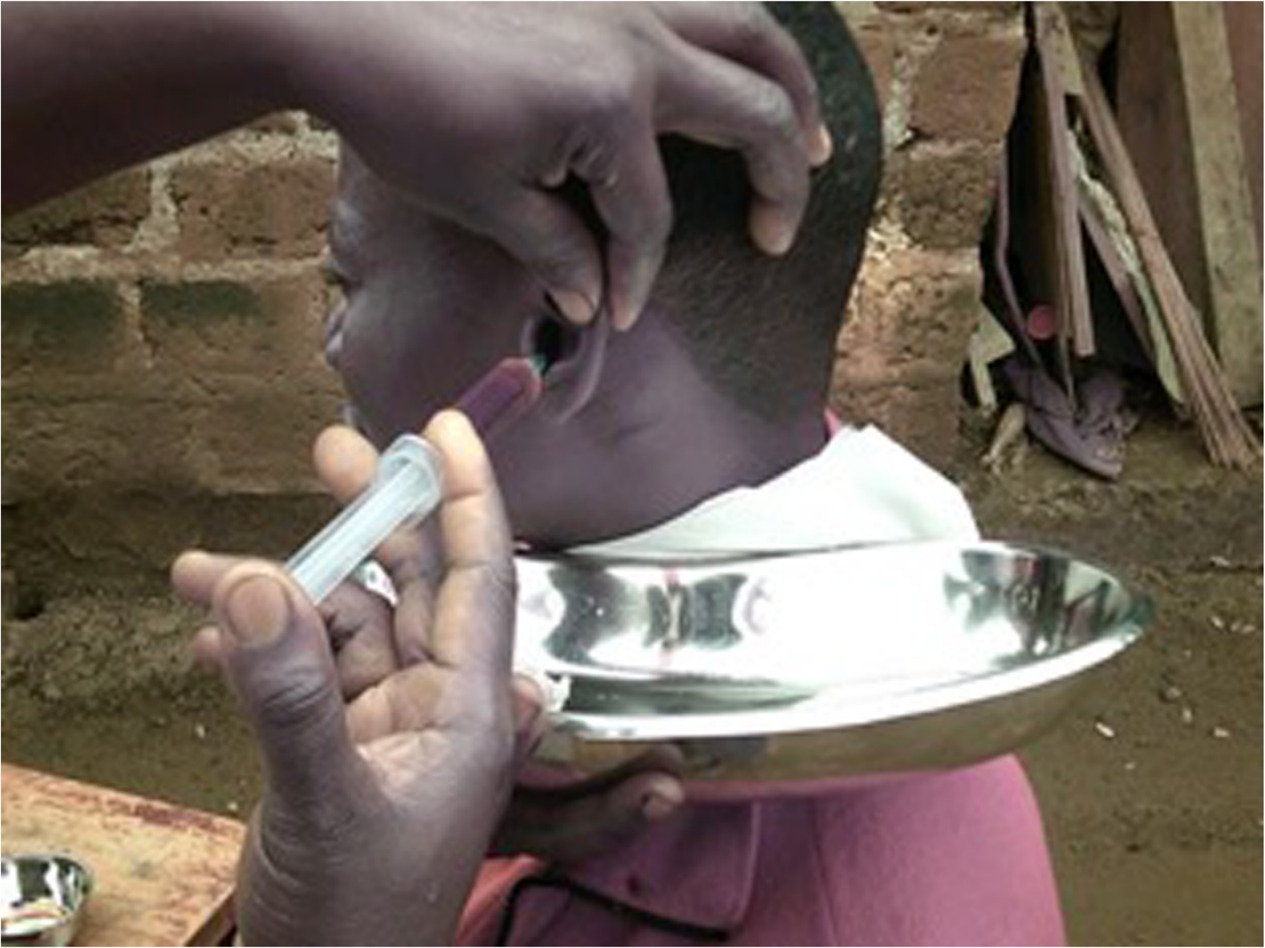
Ear Washout. A CHW carrying out an ear wash out in the community for impacted ear wax

In another photograph a CHW can be seeing using a syringe filled with sterile water mixed with iodine to wash out a patients ears in a community setting (Fig. 2):

*“The patient had wax so I was doing an ear wash out…..the project is about treating people with ear problems and what I was doing is a part of it. I wanted to show you that I am doing what I was trained to do.”* (CHW 13).

Similarly CHWs used photovoice as an opportunity to document many positive testimonies from community members, which they could then share with their supervisors to document good work. In one example a CHW captured a photograph of a female patient sat outside her house:

*“This lady said her ears were itching so I examined them and found wax. I gave her olive oil and showed her how to use it for five days, after five days I did the washout and there was a very big change…I felt so good and confident, that I know what I am doing…They (the other members of the community) all believe in me now - for instance that lady is always referring people to me. Initially people were saying this project is going to damage people’s ears but now they are saying the opposite.”* (CHW 11).

### Theme 2: there are structural and logistical barriers to delivery of ear and hearing care at the community level

Several barriers were identified regarding delivering ear and hearing care in the community. These included those related to the wider health services and those related to CHWs themselves.

One example of a CHW related barrier was community and health staff perceptions of the position and role of CHWs in society. Several CHWs stated that towards the beginning of the project they encountered suspicion or challenges from the community, since their previous roles were less focussed on health interventions, and more on health promotion initiatives. One CHW took a photograph of other CHWs at a training session and stated:

*“They just don’t think one can be trained in a month and be good at whatever they were trained in especially to do with diagnosing and treating people. They also feel what we used to do before like encouraging people to construct latrines is lower compared to treating ears and wonder how fast our status was elevated.”* (CHW 11).

Other CHWs noted that interacting with the health system may be impeded by its hierarchical structure, noted through a discussion around a photograph featuring a clinical officer at the health centre observing a patient referred to him by a CHW.

*“Now if we are here at the Health Centre, that man (the clinical officer) is like my boss, so it may not be easy for me to go and inquire from him about my patient that he worked on, he may look at it as if am trying to undermine his role.”* (CHW 12).

In terms of structural barriers related to wider health services, it was noted that many of the community members were reluctant to travel to the local health centre to seek care, given the long distances involved and uncertainty about whether they would receive treatment from the staff posted there. This was illustrated in a photograph of SN HCIII where patients sat in a busy waiting room waiting to be seen by a clinical officer:

*“People go to the health centre and find them (Health Centre Staff) feeling irritated, probably from salary delay so they decide to attend to their other businesses…. they spend the whole day in line with no one attending to them.”* (CHW 7).

Secondly, staff at the local health centre were not properly trained in how to deliver safe and effective ear and hearing care services; some CHWs reported potentially unsafe practices including the use of sharp scissors to remove foreign bodies from the ear canal. To illustrate this point a CHW captured a photograph of a patient who had come to see him after being seen by staff at a local health centre:

*“There is this one patient I referred from Nakabago and nurses here used scissors to examine her but she was not so happy about our way of examination. She said it was so painful.”* (CHW 6).

Thirdly, the local health centre was poorly equipped and often out of stock of many of the basic medications for treating ear disease, such as topical ciprofloxacin antibiotic ear drops. To illustrate this point a CHW captured a photo of an empty stock room at the local health centre:

*“Local hospitals don’t even have otoscopes and health workers use light from their phones to examine ear patients. It is by luck that they are able to identify the problem but then if one has a hole, how are they able to see it? Those who have pus are washed out using water with nothing more and after that they are not given antibiotics. Many of those health workers are not qualified so they have little or no knowledge about what they could be doing.”* (CHW 1).

### Theme 3: there are traditional beliefs in the community on the cause and therapy of ear and hearing disorders

Several discussions around the photographs highlighted traditional beliefs of the local population regarding the causes of ear disease. For example, one photograph was of a group of women attending SN HCIII for a childhood vaccination clinic. The CHW who took the photograph engaged the women in a conversation regarding causes of chronic ear discharge in children, and found that many of them believed spilled breast milk was the cause of pus in the ear.

*“Many of these women believed in ‘mabere’ (spilled breast milk causing pus in the ears) and wanted to know if it is true.”* (CHW 5).

**Fig. 3:**
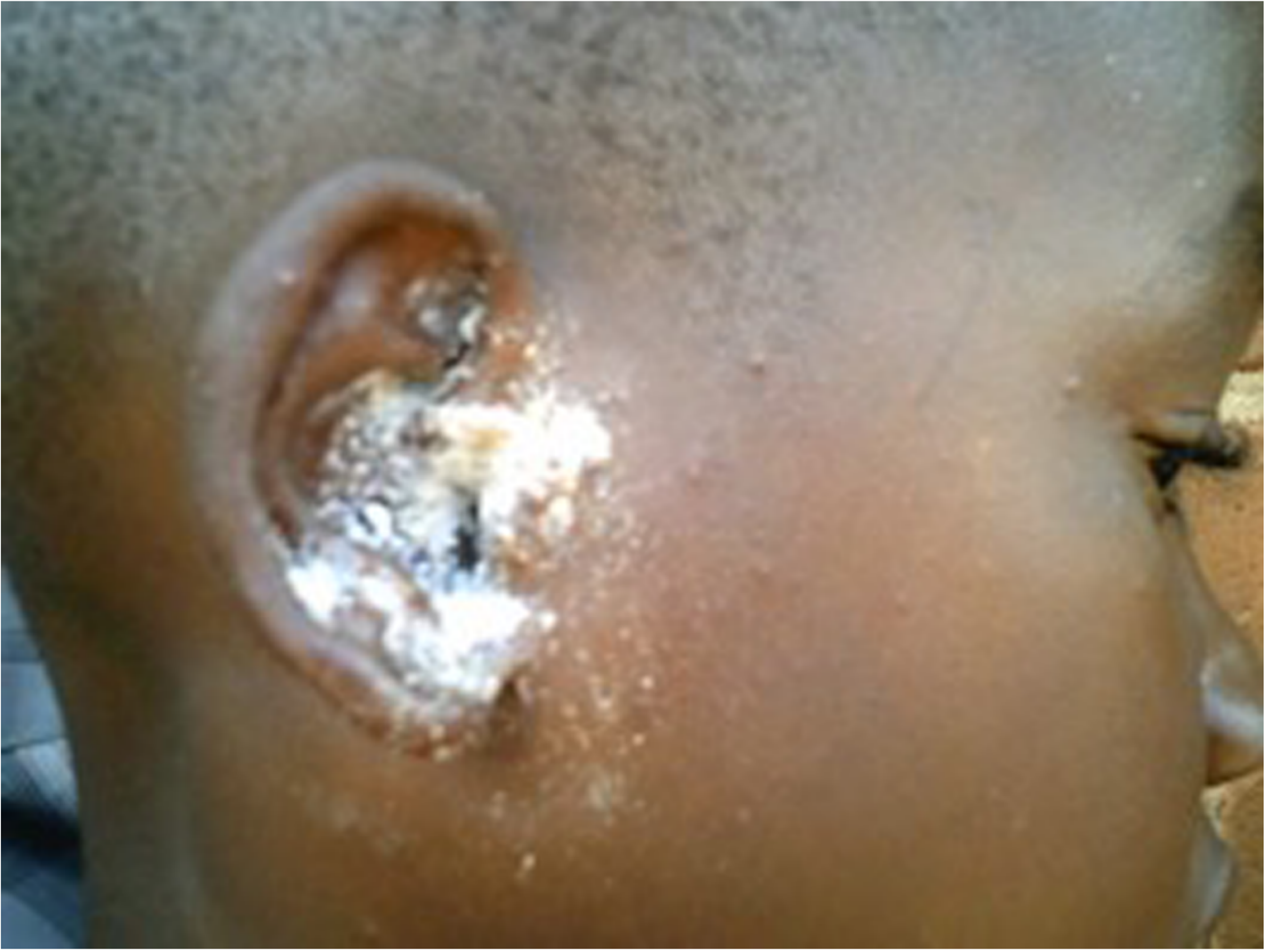
Traditional beliefs regarding treatment of ear disease. A child with roasted bone placed around the ear to treat external wounds

These discussions also highlighted local remedies to ear disease, such as putting herbs in the ear canal, and applying crushed cow bones around and in the ear canal to treat external ear wounds (Fig. 3).

*“Her mother roasted a bone, crushed it and applied it to the child’s ear as medicine… That is the treatment recommended in our communities. The condition of someone having wounds on their ears is called maddu (greed) in Luganda. Remember the origin of the word is from someone liking meat a lot and because of that, people have a belief that a bone can heal those wounds. I examined her and saw some pieces of the bone in the ear canal.”* (CHW 1).

### Theme 4: socioeconomic deprivation influences prevalence of disease and prioritisation against competing needs

**Fig. 4:**
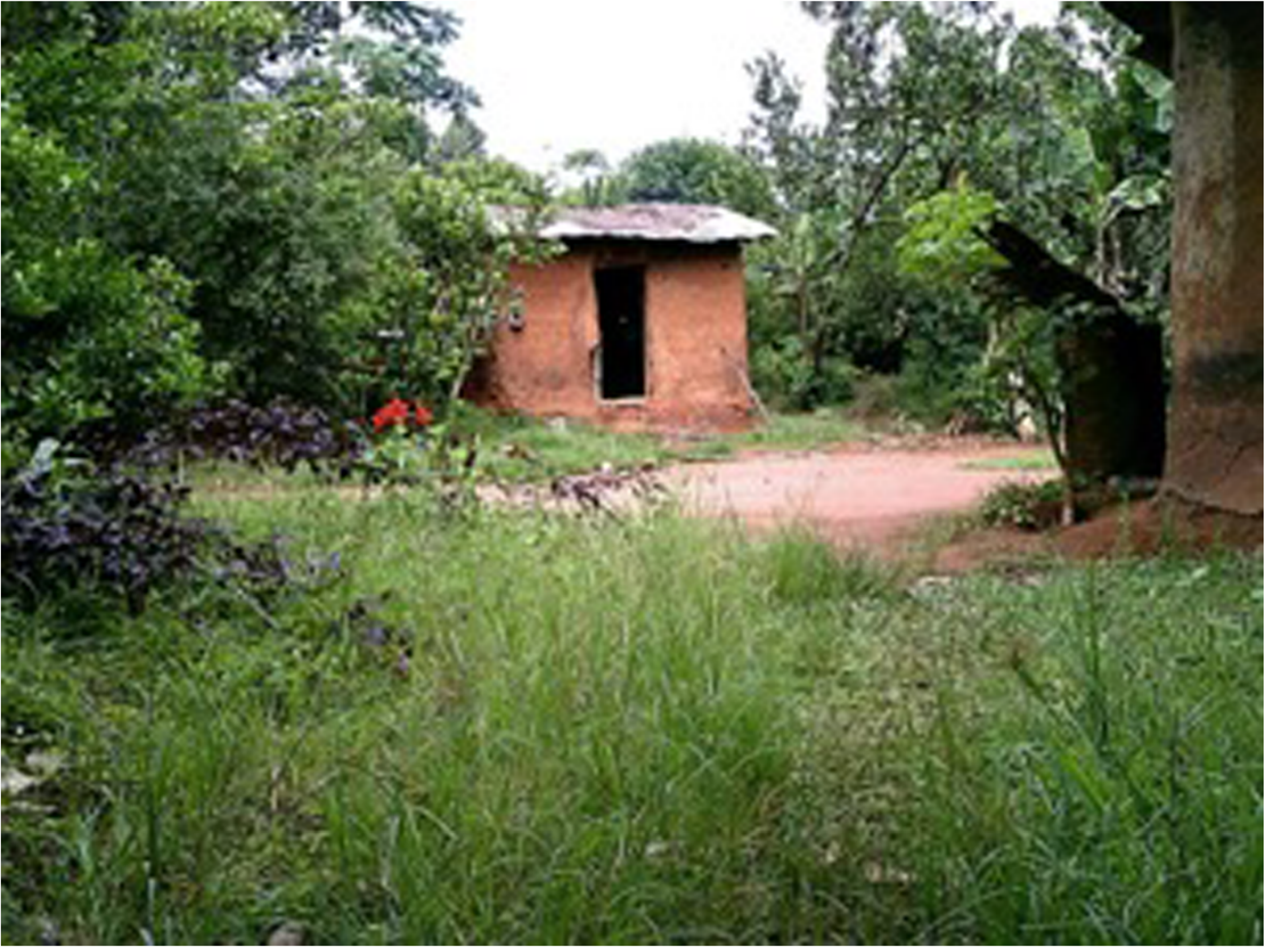
A mud brick house. A mud brick house, common for the local area, reflecting high levels of poverty in the local area

Several photos highlighted the impact of social status and income on ear and hearing health. For example, one of the CHWs captured a photograph of a mud house which many people in his village lived in and related that back to the high prevalence of ear disease he had observed (Fig. 4).

*“That is a mud house and I think you can see the state of the walls and iron sheets. They have holes in them. We have a couple of people in the community living like that. With such a house, people are likely to wake up with insects in their ears which we see many cases of. Even if you treat them they will still have them again because the cause is not being gotten rid of.”* (CHW 8).

Similarly, socioeconomic deprivation often meant that community members were unable to attend regional ENT hospitals in the cities of Mbarara or Kampala for specialist treatment. The majority of people in the area were subsistence farmers living on less than $2 USD/day, yet travelling to one of the regional referral hospitals would require taking a public bus (over 3 h one-way), paying for the medication and anaesthesia at an approximate cost of $55 USD, and finding accommodation to stay overnight. To illustrate this one of the CHWs took a photograph of a pig.

*“People are always referred to specialists like the ENT surgeons and audiologists if their problems are so complicated. Someone who owns that one pig cannot afford to see them because they are expensive and no amount of money got from that pig can pay for their treatment. I took the photo so you can see some of our problems in the community because most people who have them (pigs) own just one.”* (CHW 8).

Other CHWs suggested that the socio-economic situation of many individuals prevented them from prioritising ear disease given the high burden of other disease, and that this was compounded by the high numbers of children per household, many of who were orphans. A CHW captured a photograph of three small children who were orphans living near to them to illustrate this point:

*“Most of these children are orphans or were abandoned by their parents and now stay with their grandparents who are too old to take good care of them. Some of the grandparents or the children themselves are HIV positive and all they care about is swallowing their ARVs [anti retro-virals], so ear problems are not an issue.”* (CHW 5).

### Theme 5: CHWs can have a key role in the provision of primary ear and hearing care

Many of the CHWs identified that they have important roles to play in the delivery of ear care, including sensitising the community and raising awareness, continuing provision of treatment, prevention and referrals, and follow up of patients.

The CHWs suggested they could sensitise the community and raise awareness of ear disease through conducting community based training and hosting events at popular community locations such as churches and mosques, as well as informing community members about outreach events, such as the ear camps hosted at the local health centre. In an interview concerning a photograph depicting a CHW giving a talk to community members sat at the local health centre the CHW stated:

*“It is my responsibility because I was trained to sensitise others. When we talk to people, they may not be having the problem themselves, but they go back and let others know. As VHTs we need to organize trainings and sensitise people about such beliefs. Even if one comes across a group of three people, one can go ahead and teach them the right thing. We should also make use of any gatherings in our villages and churches after seeking permission from the village local council.”* (CHW 1).

They also noted that they were well positioned within the community to help address traditional beliefs which might further compound ear issues. For example, one lady who had suffered hearing loss for several years had used traditional-healers, but after the CHW spoke to her and gained her trust she agreed to visit the ENT specialist who attended SN HCIII:

*“I sensitised her, gained her trust, and also listened to her concerns…otherwise that lady wouldn’t have accepted to go and be seen by Dr. Daniel (ENT specialist).”* (CHW 11).

A second important area identified by CHWs as to where they could help address the burden of ear and hearing disorders was in treatment and prevention. CHWs indicated that they could continue being an important resource for providing basic treatment, making referrals where appropriate, and encouraging community members to take preventative measures to avoid ear disease.

*“It is their responsibility to seek treatment and it is the responsibility of us VHTs to treat them. We also need to sensitize them on how to prevent it in future.”* (CHW 3).

*“…for the boy’s case, he needs ENT specialists. It is still the responsibility of the VHT to refer him.”* (CHW 12).

The final role they identified for themselves was in visiting patients in the community to either assist in early detection of cases of ear disease, or to ensure they provide continuity for patients who have received treatment. CHWs illustrated this through photographs depicting them conducting household visits:

*“My role is to supervise the patients, like after every two weeks, I go back to the patient and I ask for permission from the mother so that I may look into the ear of the child again to see the condition because I want him to get well…”* (CHW 8).

### Theme 6: training, supervision and equipment is critical to successful delivery of ear and hearing care

Several of the CHWs took photographs representing programmatic factors they felt crucial to successful delivery of a primary ear and hearing care service, including the provision of diagnostic equipment, medicines, and on-going support.

*“Having that equipment readily to hand means we can do our job easier. It cuts down on the time needed to refer a patient to the health center as I can treat them immediately.”* (CHW 6).

Other CHWs inferred that having equipment helped them to gain more trust and respect from the community:

***“****Patients trust us when they see us with them.”* (CHW 7).

*“I took that photo because when I go to the field, I have to show the people the things that I use when am examining them and I tell them that even the mzungu (foreigner) who trained us is proud of the work we are doing, that is why he gave us this equipment such as we can use them on our home visits and in our community.”* (CHW 2).

Several CHWs took photographs in which they were being supervised, or were receiving on-going training as part of a workshop. Supervision was highlighted as important because CHWs felt that it created a sense of accountability, and because it helped remind them about their roles and how to perform them. This included technical skills, such as how to hold the otoscope, and non-technical skills, such as how to take consent and treat community members in a respectful manner.

*“I learnt to be patient and tolerant with community members and not to be rude at patients.”* (CHW 7).

**Fig. 5:**
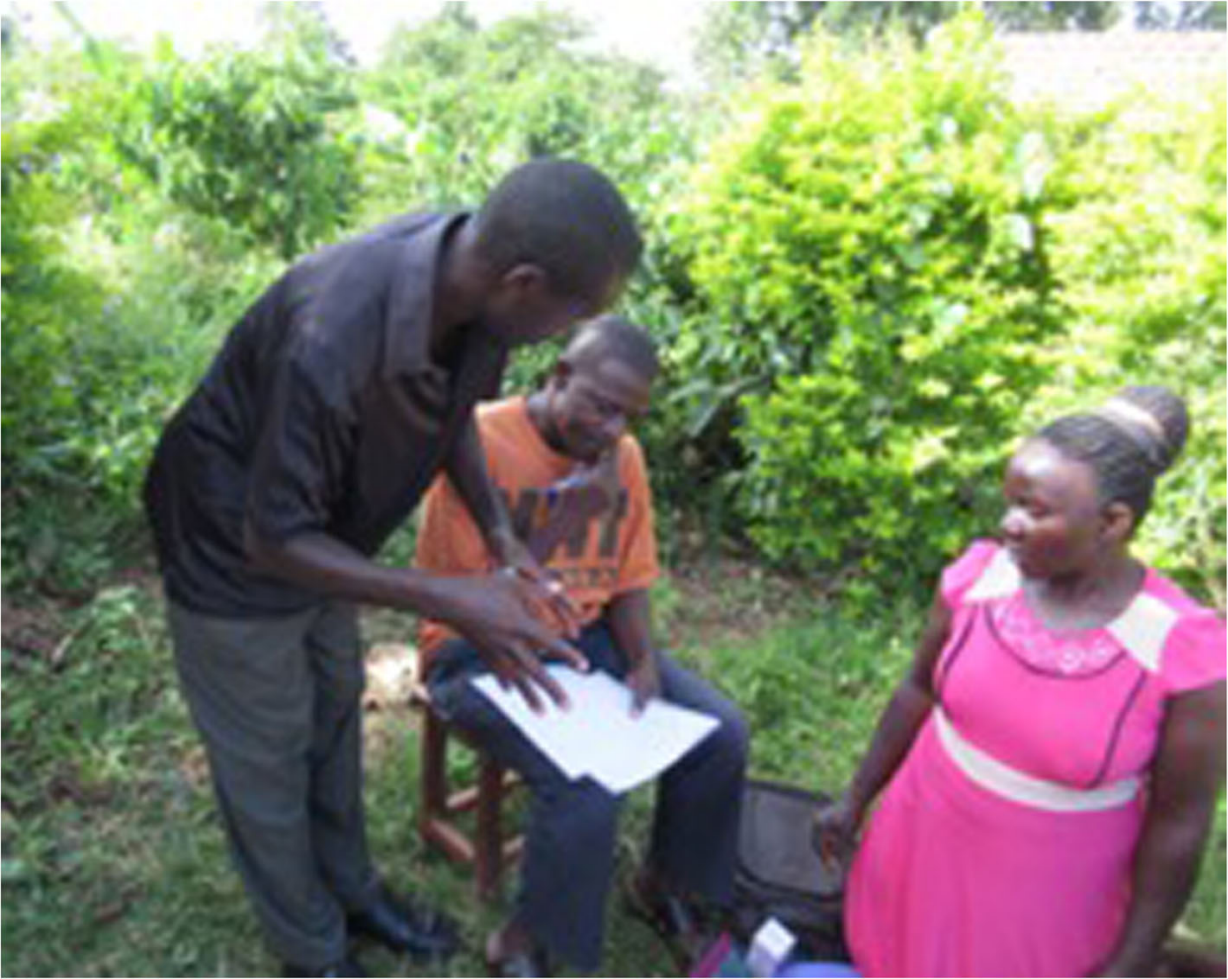
Supervision in the community. A CHW (orange t-shirt) being supervised on a home visit in the community by a peer-supervisor (black shirt)

One of the CHWs suggested that supervision not only benefitted the CHW to have their work observed, but it also helped the community member feel more secure. This was illustrated in a photograph of a CHW supervisor overseeing them conducting outreach work in a patients home (Fig. 5):

*“When patient sees that it is two of us treating them, they feel more secure. Also, two heads are better than one.”* (CHW 9).

**Fig. 6:**
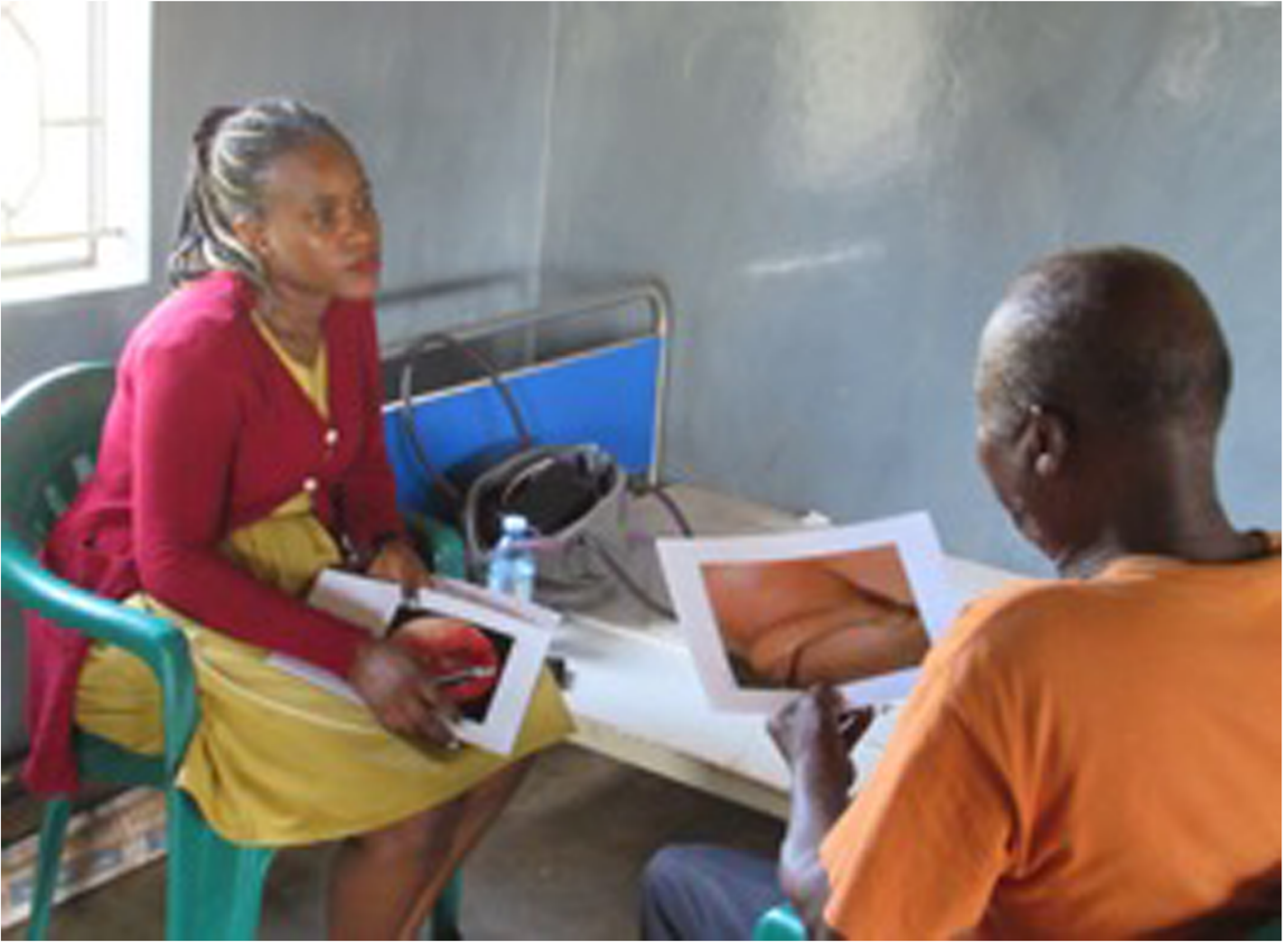
On-going training and assessment. A male CHW (orange t-shirt) being assessed by a female ENT surgeon (wearing a yellow dress) at a refresher training workshop

On-going training and assessment was also recognised as important. This was highlighted by the CHWs taking photographs of a refresher training workshop they attended (Fig. 6). The CHWs suggested that this process helped to make them aware of certain conditions they had not been taught about in detail in the original training, such as fungal ear infections, as well as ways to improve their performance and address areas of potential weakness.

*“I love evaluating myself to find out my weaknesses and then I can overcome them. I can also discover where my strength lies so that I can keep up with it or even help my fellows who may be struggling in that particular area.”* (CHW 2).

*“I was so happy about it because we were able to evaluate ourselves. They also reminded us what we had forgotten.”* (CHW 7).

CHWs suggested that supporting NGOs, such as Omni Med, have important roles in continuing to assist in CHW training and supportive supervision.

*“I think it is both my responsibility and that of Omni Med. I am responsible for making a follow up on such patients, but Omni Med provides me with the support like the equipment or a review from my supervisor.”* (CHW 12).

### Theme 7: ear and hearing disorders should be given greater priority by the government

CHWs suggested that greater attention should be given to ear and hearing disorders by government health officials, both at a national policy level by the Ugandan Ministry of Health, and at a local level by the District Health authorities. CHWs cited increased funding, greater training of health workers, and provision of supplies at government health centres as important areas to address. There was also the suggestion that the government should help place ENT specialist doctors in the community and bring specialist services closer to the community.

*“The government should allocate more funds to ear health programmes.”* (CHW 13).

*“What should be done is to bring closer the services of ear care to the communities for example instead of someone being referred to Mbarara, there should be surgeons around so that people don’t move long distances.”* (CHW 5).

They also highlighted the need for government funded programmes to supply and maintain hearing aids, given the burden of hearing loss in the community. Again this was illustrated through a photo of an individual in the community.

*“Our government should start thinking about people with such cases and make hearing aids affordable.”* (CHW 8).

### Results from the community workshop

In total 42 individuals attended the community workshop. A breakdown of attendees by stakeholder group can be found in Table 3.

**Table 3:** Attendees at community dissemination workshop

Stakeholder Group	Number of Attendees (%)
Community Health Workers	16 (38%)
Community members	14 (33%)
Non-governmental Organisation staff	4 (10%)
Local governance representative	4 (10%)
Health Facility Staff	2 (5%)
Religious leader	1 (2%)
Peer educator	1 (2%)

Breakdown of attendees at community dissemination workshop by stakeholder group.

Of the 42 attendees, 36 (86%) completed the questionnaire. Of these, 32 (32/36 = 89%) stated that they interacted with someone who they would not normally interact with and for 26 (26/36 = 72%) this was the first time they had attended a workshop where photographs were used to document community health issues. All but one participant stated that they found the photographs to be very useful (13/36 = 36%) or extremely useful (22/36 = 61%) in helping them to understand community-based ear care. Results of the questionnaire are shown in Table 4.

**Table 4:** Likert scale ratings on questionnaire at the community dissemination workshop

Rating	1	2	3	4	5
1. How clear were the objectives of the workshop?	0%	0%	0%	31% (*n* = 11)	69% (*n* = 25)
2. How well organised was the workshop?	0%	0%	3% (*n* = 1)	39% (*n* = 14)	58% (*n* = 21)
3. How useful were the photographs in helping you understand community-based ear care?	0%	0%	3% (*n* = 1)	36% (*n* = 13)	61% (*n* = 22)
4. How likely are you to use the knowledge of community ear care issues observed today in your future work?	0%	0%	11% (*n* = 4)	28% (*n* = 10)	61% (*n* = 22)

For questions 1 and 2: 1 = Very poor; 2 = Poor; 3 = Fair; 4 = Good; 5 = Very good. For question 3: 1 = Not useful at all; 2 = Slightly useful; 3 = Moderately useful; 4 = Very useful; 5 = Extremely useful. For question 4: 1 = Extremely unlikely; 2 = Unlikely; 3 = Neutral; 4 = Very likely; 5 = Extremely likely.

A common theme that arose during the open discussion at the end of the workshop was the desire for government officials to be in attendance. Participants felt this representation was important for the long-term sustainability of the project. The representatives of the health centre wished for the project to be expanded, and felt that this forum was a good opportunity to display the high burden of disease and to raise awareness, but questioned the non-attendance of the district government health officials since it was ultimately them who would lobby for increased funding from the central government. The workshop was filmed by the video journalist and broadcast that evening on the national news channel, Bukedde TV.

## Discussion

This is the first study to explore CHW perceptions, barriers, and enablers to in-community delivery of ear and hearing care. We found the photovoice method provided a variety and depth of opinion from participants and allowed us to gain important first-hand perspectives from CHWs themselves.

It is clear that several contextual factors impact the equity of a CHW led ear care programme in this context, including socio-cultural practices and beliefs, living circumstances, and health system factors [26]. These factors can be categorised into demand- or supply side determinants [27]. Demand-side determinants influence health-seeking behaviour and health service access (for example, the acceptability of a health service in a community), whereas supply-side determinants are related to health-system factors which are influenced by policy (e.g. CHWs being only trained to deal with maternal and child health issues, or not having ENT specialist doctors based in the community) [6]. It has been suggested that these factors are more likely to negatively impact poor and vulnerable groups due to accessibility issues, cultural barriers, and lack of information preventing them benefitting from public health interventions such as this [28]. Here we will explore the perceptions, barriers, and enablers to in-community delivery of ear and hearing care that were developed through the use of photovoice, viewed through a health equity lens.

### Perceptions

Our study shows that photo documentation was perceived by CHWs as a mechanism to convey health information and learning. Similarly a study from Kenya found that CHWs frequently posted photographs between themselves and their supervisors in a closed WhatsApp group to document their work and obtain feedback [29]. Other authors have suggested that visual imagery may help to convey important health messages by helping to create a story and invoke the emotions of the learner [30] and by assisting long-term recall of cases [31]. Exploring how images could be used in health programs could be the subject of future studies. From the perspective of the CHWs the use of photographs to explore a topic of community importance was generally well received, however some challenges were encountered which have been documented in the limitations section of this paper.

Importantly we also found that CHWs perceived a large local burden of ear and hearing disease. In particular there was a high prevalence of chronic suppurative otitis media, which has previously been reported in Uganda [32], as well as other LMICs [33]. The CHWs themselves recognised the impact of socio-economic deprivation on prevalence of ear disease and several of the photographs documented advanced or persistent disease. An encouraging finding was that the CHWs perceived that they could play a significant role in tackling this burden of disease. For simple cases they appeared able and willing to provide management, and for more complex cases to refer to specialist care. Many CHWs captured photographs of people they had successfully treated in the community, to document positive testimonies from patients, or to show their supervisors the work they had completed. From an equity perspective, this particular model holds promise in extending the reach of ear and hearing care services, especially to those in rural and remote settings such as this. This mirrors findings from previous studies, which suggest that CHW programmes more broadly can help promote equity in access to health services by reducing inequities such as location and socio-economic barriers [34]. However, despite the perceived improvements in access to ear care services, complex challenges remain when considering supply and demand dimensions to this model.

### Demand-side barriers

Several demand-side barriers were identified by CHWs including financial, structural, logistical, and socio-cultural impediments to the community in accessing ear and hearing care.

There was reluctance from many community members to travel to the local or regional health centre to seek care, given the long distances and costs involved, uncertainty about whether they would receive high quality treatment from the staff posted there, and competing health needs such as disease due to HIV. These factors mirror findings from a study in Malawi, where of 150 children referred to specialist ear and hearing services, over 97% failed to attend citing a lack of finance, transport, and/or understanding about the importance of the referral [35]. This questions if addressing ear and hearing disorders is better integrated into broad ranging health services delivered by CHWs, in contrast to vertical, disease specific approaches which have been the mainstay of community health interventions in the past [36]. From an equity perspective it will be important to consider community mobilisation and sensitisation about such services in order to maximise potential demand, as well as consider alternative, individualised approaches for delivery of care in certain groups e.g. those in very rural areas.

A small number of CHWs also reported that when they tried to perform a more clinical role, which includes diagnosis and management of disorders, they were met with resistance by some community members and by staff at the local health centre. Involving community leaders such as village chairpersons to advocate for CHW led care, as well as engagement with other local and regional health providers, may help to increase community acceptability, and is an important equity consideration for programme planners.

A third demand-side barrier was the adoption of traditional medicine for ear and hearing disorders in this region of Uganda. Traditional beliefs were complex and wide-ranging (from the cause of ear disease, through to traditional forms of treatment), and rooted in cultural and linguistic traditions. For example we discovered that the Luganda word *“maddu”* meaning *“greed”* is used to describe external ear wounds, and thus the local treatment was to use crushed cow bone in order to *“satisfy the greed”*. Traditional medicine has been used for hundreds of years in Uganda and up to 80% of the population use traditional medicine for primary health care [37]. Use of traditional medicine in ear and hearing care is documented in other LMICs, for example an ear discharging pus may be treated with oil or honey in Nigeria [38], or oil, pastes or urine in Nepal [39]. Approaches delivered by external actors based upon biomedical models might be met with suspicion and resistance, and risk a cultural-ideological clash [40]. Programs need to be sensitive to and explicitly address traditional beliefs, and may benefit from engagement in a pragmatic manner; something which has been reported in other settings [41, 42]. Working with traditional healers and inviting them to be part of initial training workshops may be one mechanism to build trust and to navigate potential areas of tension in a culturally appropriate manner.

### Supply-side barriers

Considering supply-side dimensions, CHWs identified the lack of human resources, or infrastructure both at the primary and secondary care level. This is a common finding in LMIC settings [5], and evidences the importance of developing CHW programs in tandem with appropriate infrastructure, and with links to services for specialist ear and hearing care [43]. The infrastructure CHWs identified as necessary to support them in their role included provision of equipment, on-going training, and supervision. These have been recognised as essential components of successful community health worker programmes more generally [44]. Provision of equipment helped CHWs provide care at the point of consultation, which in the context of this study was often in community members’ homes in remote communities. Supervision and refresher training have been poorly documented in previous studies and should be considered at an early stage in the design of programmes [45]. Future work could explore blended strategies of supervision combining in-person home visits (which appeared to be acceptable both from the CHW and community perspective), and remote support using mobile technologies.

A second supply-side barrier is that the cost of specialist or surgical care is also significant. Although treatment is theoretically free-of-charge at government health centres in Uganda, in reality patients often have to purchase their own medicines, anaesthetics, and operating consumables due to inadequate supplies [46, 47]. A 2017 study found that 31% of patients at a major regional referral hospital in the southwest of Uganda faced catastrophic expenditure to access surgical care [47]. From an equity perspective it is therefore important to note that even if CHW services are provided in the community, payment for specialist surgical services can continue to represent a barrier. Mobile ear camps where surgery is performed in the community have been described in Nepal and India [48, 49], and represent one way to bring surgical care closer to the community; however, it is important to caveat that these need to be supported with adequate resources and well trained staff to maximise chances of positive impact. Although CHWs are able to play important roles in awareness raising, screening and basic management of ear disease and hearing loss in the community, ultimately more advanced and complex cases require specialist input. Investment in such services is therefore imperative.

### Enablers

CHWs identified that NGOs and foreign agents were critical in providing the equipment and infrastructure for training and tools for the delivery of ear and hearing care in this program. Although at present external agents can play an important role in initiating services where none exist [43], the ultimate solution must be locally led and locally sustainable.

CHWs also recognised the importance of local and national government in providing the funding and support to enable ear and hearing care. In a 2013 survey by the WHO, Uganda stated it had a national plan for ear and hearing care [50]. Other external agencies have worked to develop relevant services in Uganda [51]. However, our experience suggests that at present the infrastructure for ear and hearing care in Uganda remains rudimentary. We found the community workshop to be a useful method in bringing together local and regional stakeholders, allowing them to interact with individuals they would not normally have the chance to meet, and to discuss ear and hearing care which may not have otherwise been on their agenda. Other authors have noted the potential benefits of community workshops to leverage community and local engagement and action; but the existing literature in photovoice studies often fails to document key details on how images are exhibited, details of the attendees, and participant perceptions [52]. Community workshops could be an important mechanism to engender “bottom-up” action, to complement “top-down” strategies from international agencies such as the WHO [53].

### Study limitations

From a reflexivity standpoint the position of those conducting interviews can influence results in qualitative studies, and here the fact that the Principle Investigator (PI) of the project was a white British researcher potentially impacted the acceptability of the method. For example, two of the CHWs stated that a small number of participants refused to grant consent to have their photo taken, out of fear that the PI would use the photos for personal or financial gain:

*“They believe bazungus will use their photos to get money from developed countries yet they personally gain nothing.”* (CHW 10).

*“A small number of people declined and kept saying the muzungu who asked us to take them was going to use their photos to solicit for funds from developed countries.”* (CHW 6).

Other CHWs commented on the perceived respect garnered from community members when they were seen with a white individual.

*“This project earns me respect from people since I am moving with a white person. It shows I am on a different level now. Also, people are always positive they will get cured once there is a white person involved and trust us a lot more that we know what we are doing…Whites are highly respected in our society so moving with them automatically earns one respect. Much as we don’t have high academic qualifications, they consider us powerful if they see us moving with him.”* (CHW 9).

These observations are important so that future researchers are aware of the issues their own position can have on the research process and how the positionality of a research team can impact on the study findings, especially in a post-colonial setting such as Uganda.

A further limitation of this study was that we did not get equal participation from stakeholders. In particular, many invited policy makers did not attend the community workshop, and financial restrictions prevented us opening the workshop to a greater number of individuals the general public. We were also unable to obtain precise figures on the number of people in each household that the CHWs served. This was partly due to migration of individuals, recent deaths and births and a lack of a recent formal household survey. Finally, not everyone who attended the workshop completed a feedback form.

## Conclusion

This study adds to the growing literature on the role of CHWs in delivering primary ear and hearing care in low resource settings [8], and is the first to document the perspective of CHWs in such a role. We found a participatory approach using photovoice captured important socio-cultural challenges which may have been missed through traditional methods of enquiry; such as researcher led one-to-one interviews.

Our findings show that CHWs perceived a large burden of ear and hearing disorders, but also recognised they could play an important role in tackling such an issue. However, in order to maximise equity of such a programme, demand- and supply-side barriers must be addressed. Barriers identified in this context included provision of adequate equipment, training, and supervision to CHWs; financial, logistical and psychological impediments to community participation; and prevalent use of traditional medicine. Support from NGOs, as well as from local and regional government or other organisations were viewed as important enablers.

The complex and diverse themes highlighted here may help guide interventions to ensure they are responsive to the needs of CHWs delivering ear and hearing care in the community, as well as help us to understand the contextual factors of such a programme which can impact health equity within the target population. Future studies could further explore how issues identified through the capture and analysis of photographs in the community may engender community (or wider) action to tackle such issues.

## Data Availability

The datasets generated and analysed during this study are not publicly available due to IRB requirements regarding data privacy and image rights, but are available from the corresponding author on reasonable request.

## Abbreviations

CHW: Community Health Workers
DHO: District Health Office
ENT: Ear, Nose and Throat
IRB: Investigational Review Board
LMIC: Low- or- middle income
NGO: Non-governmental organization
RA: Research Assistant
SN HCIII: Seeta Nazigo Health Centre III
USD: United States Dollars
VHT: Village Health Team
WHO: World Health Organization
